# Apolipoprotein B in cardiovascular risk assessment: beyond traditional lipid markers

**DOI:** 10.3389/fcvm.2026.1857230

**Published:** 2026-09-30

**Authors:** Lingling Li, Yixuan Feng, Xiaoya Li, Zongliang Yu, Changxin Sun, Lanqing Hu, Kaidong Zhou, Xinyi Liu, Yongfang Yuan, Liang Li, Min Wu, Longtao Liu

**Affiliations:** 1 Department of Cardiology, Guang'anmen Hospital, China Academy of Chinese Medical Sciences, Beijing, China; 2 National Clinical Research Center for Chinese Medicine Cardiology, Xiyuan Hospital, China Academy of Chinese Medical Science, Beijing, China; 3 Graduate School, Beijing University of Chinese Medicine, Beijing, China; 4 Geriatrics Dept, Zhongshan Hospital of Traditional Chinese Medicine, Zhongshan, China

**Keywords:** apoB, atherosclerotic cardiovascular disease, cardiovascular disease risk, lipid biomarkers, therapeutic targets

## Abstract

Apolipoprotein B (ApoB) serves as the structural backbone of triglyceride (TG)-rich lipoproteins and low-density lipoprotein (LDL) particles and plays a key role in lipid transport. A growing body of evidence indicates that circulating ApoB levels are closely associated with the development of cardiovascular diseases such as coronary atherosclerosis, and ApoB may be one of the most accurate markers for assessing atherosclerotic cardiovascular disease (ASCVD) risk. This review discusses the limitations of conventional lipid measures, including low-density lipoprotein cholesterol (LDL-C), non-high-density lipoprotein cholesterol (non-HDL-C), and triglycerides (TG), and systematically examines the advantages of ApoB as an alternative marker for cardiovascular risk assessment, as well as its independent predictive value. In addition, this review discusses the clinical utility, potential advantages, and challenges associated with ApoB, as well as the effects of currently available lipid-lowering therapies on ApoB and recent advances in the development of ApoB-targeted therapies, with the aim of informing more precise strategies for the prevention and treatment of cardiovascular disease.

## Graphical Abstract

**Figure:**
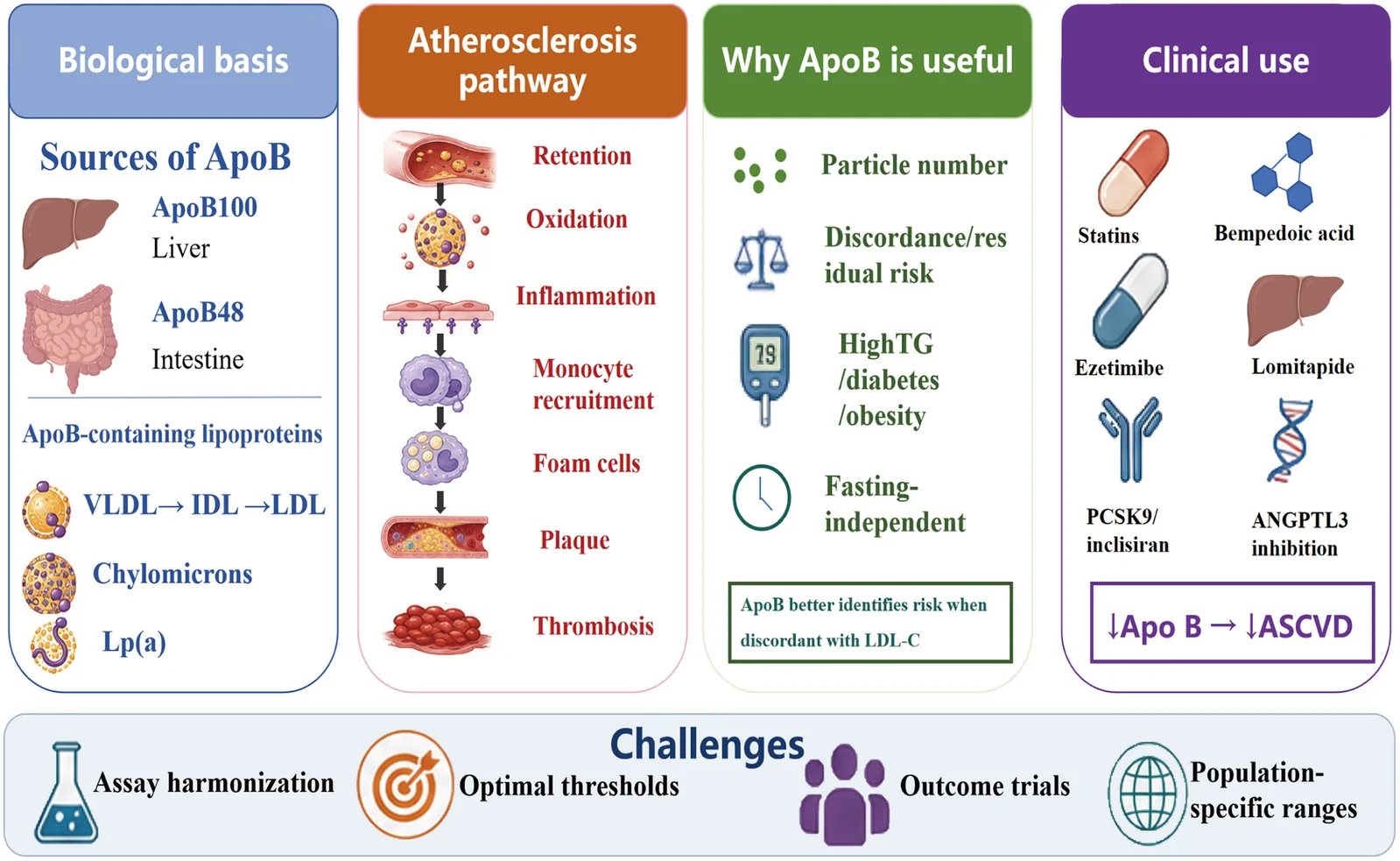
Overview of apolipoprotein B (ApoB) in cardiovascular risk assessment. ApoB, Apolipoprotein B; VLDL, very-low-density lipoprotein; IDL, intermediate-density lipoprotein; Lp(a), lipoprotein(a); LDL-C, low-density lipoprotein cholesterol; ASCVD, atherosclerotic cardiovascular disease; PCSK9, proprotein convertase subtilisin/kexin type 9; ANGPTL3, angiopoietin-like protein 3.

## 1 Introduction

Cardiovascular disease (CVD) is a leading cause of morbidity and mortality worldwide (1). Coronary heart disease (CHD) accounts for approximately half of all CVD cases and cardiovascular deaths (2). Dyslipidemia is a major risk factor for the development of CVD and CVD-related mortality (3). Triglycerides (TG), low-density lipoprotein cholesterol (LDL-C), and high-density lipoprotein cholesterol (HDL-C) have long been the primary targets of lipid-lowering therapy (4, 5). Among these, LDL-C is the primary target of lipid-lowering therapy for the management of hypercholesterolemia and chronic coronary disease (6, 7). Studies have shown that residual risk persists even when standard lipoprotein targets are achieved (8). Accordingly, the potential value of other lipoproteins for cardiovascular risk prediction has received increasing attention.

Apolipoprotein B (ApoB) is a key structural component of atherogenic lipoproteins and is present in all circulating lipoprotein particles except HDL. Retention of ApoB within the arterial wall promotes the development and progression of atherosclerosis (9). Studies have shown that ApoB is a more accurate predictor of cardiovascular mortality risk than LDL-C (10, 11). Because the cholesterol content carried by individual atherogenic lipoprotein particles varies substantially, cholesterol-based measures such as LDL-C and non-HDL-C do not fully reflect the number of circulating atherogenic lipoprotein particles. In addition, phospholipids and ApoB within these particles can exert proatherogenic effects after oxidative modification. Therefore, compared with LDL-C and non-HDL-C, ApoB provides a more direct measure of atherogenic lipoprotein particle burden and has greater predictive value for cardiovascular risk assessment (12–14).

In this review, we describe the molecular and biological characteristics of ApoB and methods for its measurement, summarize its predictive value for CVD as an alternative marker, and discuss the clinical implications and challenges associated with its use. We also review current progress in the development of novel therapies targeting ApoB.

## 2 Molecular and biological characteristics of apoB

Apolipoprotein B (ApoB) is the major structural protein of triglyceride-rich lipoproteins and exists in two isoforms according to the site of synthesis: ApoB48, which is synthesized in the intestine, and ApoB100, which is synthesized in the liver. Although ApoB48 and ApoB100 are encoded by the same APOB gene, they have distinct physiological functions in lipid metabolism. Studies have shown that both ApoB48 and ApoB100 are derived from the same APOB gene located on the short arm of human chromosome 2 at 2p24.1. The APOB gene comprises 29 exons and 28 introns (15). Various mutations in the APOB gene can disrupt normal lipoprotein synthesis and metabolism. Some pathogenic variants may even lead to premature CVD and substantially increase the risk of death (16).

ApoB100, one of the largest proteins encoded by the human genome, is the major structural and functional component of LDL and serves as a ligand for the LDL receptor (LDLR) (17, 18). ApoB100 is a glycoprotein with a molecular mass of approximately 550 kDa and serves as the major apolipoprotein component of several non-HDL, contributing to their structure and function. This lipoprotein family includes very-low-density lipoprotein (VLDL), intermediate-density lipoprotein (IDL), LDL, and other specific lipoprotein particles (19). Most ApoB100 is secreted by the liver and incorporated into large TG-rich VLDL particles (19). As a specific marker of intestinal chylomicrons, ApoB48 has distinct biological characteristics. Its amino acid sequence corresponds to the N-terminal 48% of the ApoB100 sequence (20). ApoB48 is synthesized in the intestine and is found exclusively in chylomicrons (CMs) and chylomicron remnants (21). Each CM and chylomicron remnant (CM-R) particle contains a single ApoB48 molecule (22).

ApoB100 is a glycoprotein composed of 4,536 amino acid residues and contains multiple hydrophobic domains that serve as lipid-binding regions, as well as binding sites for LDLR and heparin-like molecules. ApoB100 comprises five structural domains: *βα*1, *β*1, *α*2, *β*2, and *α*3 (23, 24). ApoB100 assembly occurs in the liver and is mediated by the first 884 amino acid residues at its N-terminus, with the involvement of microsomal triglyceride transfer protein (MTP). MTP plays a key role during the ribosomal synthesis of ApoB. Specifically, MTP transfers TG and cholesteryl esters to nascent ApoB particles, facilitating their initial lipidation, after which these particles are translocated into the endoplasmic reticulum (ER) lumen (25, 26). ApoB100 plays a key role in the hepatic assembly and production of VLDL. Each VLDL particle contains a single ApoB100 molecule, thereby influencing the production of VLDL and TG by hepatocytes (27). ApoB48 corresponds to the N-terminal portion of ApoB100 and consists of 2,153 amino acids, representing a 48% truncated form of ApoB100. It is generated through intestine-specific RNA editing and shares the same folding domains as the corresponding N-terminal region of ApoB100 (28). ApoB48 lacks the LDLR-binding domain and therefore serves as the structural scaffold of chylomicrons, which mediate the absorption and transport of dietary lipids (29). Several studies have shown that fasting ApoB48 concentrations can effectively reflect postprandial lipid metabolism (30, 31). Based on these findings, fasting ApoB48 levels may serve as a potential biomarker for atherosclerotic diseases, with particular clinical relevance to cardiovascular disease and ischemic stroke (32).

## 3 Measurement and standardization of apoB

Although several guidelines have recognized ApoB as one of the most accurate lipid markers for assessing atherosclerotic cardiovascular disease (ASCVD) risk and treatment response, and have acknowledged that ApoB measurement is more standardized and accurate than the estimation of LDL-C and non-HDL-C, some guidelines continue to question its reliability (33, 34). Current methods for measuring ApoB mainly include immunoturbidimetry, enzyme-linked immunosorbent assay (ELISA), and liquid chromatography–tandem mass spectrometry (LC–MS/MS). Immunoturbidimetry is based on changes in turbidity resulting from antigen–antibody reactions and is suitable for high-throughput clinical testing, with good reproducibility and amenability to automation (35). Analysis of purified lipoproteins by Chen et al. (35) showed that immunoturbidimetry can accurately measure ApoB in normal human plasma, with good dilution linearity and spike recovery. ELISA provides high specificity and sensitivity for ApoB measurement, and a monoclonal antibody-based ELISA has been proposed as a candidate reference method for ApoB measurement (36). In addition, mass spectrometry has emerged in recent years as a high-precision method that enables simultaneous quantification of multiple apolipoproteins and may further improve the standardization of ApoB measurement (37, 38).

ApoB measurement offers several advantages in clinical practice. It is highly standardized; in 1994, the International Federation of Clinical Chemistry (IFCC) and the World Health Organization (WHO) approved a standardized protocol for ApoB measurement and introduced uniform reference materials, thereby minimizing interlaboratory variability (39). The results are unaffected by fasting or nonfasting status and are also not influenced by hyperlipidemia, with no need for additional calculations (40). In contrast, calculated LDL-C becomes less reliable at high TG levels, and the conventional Friedewald equation shows substantial errors when TG levels exceed 4.5 mmol/L, limiting its applicability (41). Although calculation of non-HDL-C avoids errors associated with the TG conversion factor (42), its calculation still depends on HDL-C measurement, which may be affected by elevated TG levels and matrix effects associated with dyslipidemia (40).

## 4 Evidence for apoB in cardiovascular risk assessment and clinical management

Studies have shown that ApoB is a more accurate marker of cardiovascular risk than TC or LDL-C (43). Prospective observational studies have confirmed these preliminary findings (44). The key evidence supporting the superiority of ApoB over traditional lipid markers for cardiovascular risk assessment is summarized in Table 1. A multicenter prospective cohort study found that, among young adults with discordantly low LDL-C and high ApoB levels, the risk of coronary artery calcification (CAC) was significantly increased (OR ≈ 1.55–2.28), and ApoB showed a dose–response association with CAC in middle age (45). Allan D Sniderman et al. (46) further validated the discordance model and found that ApoB provided important predictive information for incident ASCVD events. ApoB consistently emerged as a more direct and accurate predictor of risk, whereas LDL-C, non-HDL-C, and TG were not adequate clinical surrogates for ApoB. In a large, prospective, community-based cohort study, Hwang et al. (47) evaluated the clinical relevance of ApoB and found that ApoB outperformed conventional lipid measures, including LDL-C, in predicting future CVD events. Zhang et al. (48) found that ApoB more accurately predicted long-term cardiovascular events in patients with coronary atherosclerosis and significantly outperformed conventional lipid measures in both the AS group and the CHD subgroup. Su et al. (49) analyzed data from the China Health and Nutrition Survey and found that, during 6 years of follow-up, individuals with high ApoB but low LDL-C or non-HDL-C had a significantly increased risk of CVD. Among these lipid measures, ApoB showed the strongest association with CVD risk and was more strongly associated with CVD than conventional lipid measures. Other studies have also shown that ApoB better reflects residual risk during lipid-lowering treatment than LDL-C, suggesting that it may be a preferred marker for risk assessment during therapy (50). A cohort study of 36,460 Chinese patients with coronary artery disease found that baseline ApoB levels were linearly associated with an increased risk of long-term all-cause mortality and showed greater prognostic value than LDL-C (51). Marianne Benn et al. (52) conducted a prospective study and found that ApoB predicted ischemic cardiovascular events more accurately than LDL-C, suggesting that measurement of ApoB may improve the prediction of future ischemic cardiovascular events. In a cohort study of 13,015 statin-treated patients from Copenhagen, discordance analyses showed that ApoB was a more accurate marker of all-cause mortality risk than LDL-C or non-HDL-C. In addition, ApoB was a more accurate marker of myocardial infarction risk than LDL-C (10). Verena Zuber et al. (53) used high-throughput multivariable Mendelian randomization analysis and identified ApoB as the leading factor among several correlated lipid measures, suggesting that the atherogenic lipoprotein particle burden reflected by ApoB may be an important lipid determinant of coronary artery disease (CAD) risk. Chao Fu et al. (54) performed a Mendelian randomization analysis to investigate the causal relationships between the ApoB/ApoA-I ratio and cardiometabolic disease (CMD) and its associated risk factors. They found significant causal associations between the ApoB/ApoA-I ratio and CMD and its risk factors, suggesting that the ApoB/ApoA-I ratio may serve as a potential marker for identifying individuals at risk of CMD. Richardson et al. (55) found in a multivariable Mendelian randomization analysis including ApoB, LDL-C, and TG that the associations of LDL-C and TG with CHD were markedly attenuated after adjustment for ApoB, whereas the association of ApoB remained relatively stable. These findings support the possibility that the number of atherogenic lipoprotein particles reflected by ApoB plays an important role in the development of CHD. However, because each ApoB-containing lipoprotein particle carries a single ApoB molecule, and ApoB is highly correlated with both lipoprotein particle number and lipid composition, current Mendelian randomization analyses cannot fully distinguish the direct effect of the ApoB molecule itself from that of the number of ApoB-containing lipoprotein particles. Therefore, these findings suggest that the atherogenic lipoprotein particle burden reflected by ApoB may be closely associated with CHD risk, but they do not establish an independent causal effect of the ApoB molecule itself.

**Table 1:** Evidence supporting apoB as a superior cardiovascular risk marker,.

Study design	Study population	Key finding	Superiority over traditional markers
Research review	General population	ApoB has higher prognostic accuracy for cardiovascular risk than TC or LDL-C	ApoB > TC, LDL-C
Prospective observational study	General population	Confirmed ApoB's superior prognostic value	ApoB superior to conventional lipid markers
Multicenter prospective cohort study	Young adults	Low LDL-C + high ApoB linked to higher coronary artery calcification risk (OR≈1.55–2.28); ApoB shows dose-response with coronary artery calcification	ApoB better reflects risk in LDL-C/ApoB discordant individuals
Clinical validation study	General population	Validated discordance model; ApoB predicts new ASCVD events	ApoB cannot be replaced by LDL-C, non-HDL-C, TG
Large prospective community-based cohort	General population	ApoB better predicts future CVD	ApoB > LDL-C
Cohort study	Coronary atherosclerosis & CHD patients	ApoB more accurately predicts long-term cardiovascular events	ApoB > traditional lipid markers (in AS & CHD subgroups)
China Health and Nutrition Survey (6-year follow-up)	Chinese general population	Elevated ApoB + low LDL-C/non-HDL-C associated with higher CVD risk	ApoB has strongest CVD risk association
Cohort study	Lipid management patients	ApoB better reflects residual cardiovascular risk	ApoB > LDL-C (residual risk assessment)
Cohort study	36,460 Chinese CAD patients	Baseline ApoB linearly correlates with long-term all-cause mortality	ApoB > LDL-C (prognostic prediction)
Prospective investigation	General population	ApoB better predicts ischemic cardiovascular events	ApoB > LDL-C
Copenhagen cohort study	13,015 statin-treated patients	ApoB more accurately predicts all-cause mortality & myocardial infarction risk	ApoB > LDL-C, non-HDL-C
Multivariate Mendelian randomization	General population	ApoB is the primary lipid determinant of CAD	ApoB is the most critical lipid marker for CAD
Mendelian randomization	General population	ApoB/ApoA1 ratio has robust causal link with CMD	ApoB/ApoA1 ratio is a valid CMD risk indicator
Multivariate Mendelian randomization	General population	ApoB is the most critical factor for CHD	ApoB > LDL-C, HDL-C, TG

## 5 Role of apoB in the pathogenesis of cardiovascular disease

Deposition of ApoB-containing lipoprotein particles within the arterial wall is a key driver of atherosclerosis. This pathological process persists throughout the course of the disease, from the formation of early fatty streaks to the eventual development of vulnerable plaques (9, 56). ApoB-containing lipoproteins are retained within the arterial wall through interactions with proteoglycans, where they subsequently undergo oxidative modification and trigger inflammatory responses. These processes drive atherosclerosis by promoting key pathological events, including monocyte recruitment and macrophage foam cell formation. This “retention–oxidation–inflammation” cascade constitutes a central molecular mechanism underlying the development of atherosclerosis (57). Currently, LDL-lowering therapy, a first-line treatment for atherothrombotic cardiovascular disease, is based primarily on reducing circulating ApoB-containing lipoproteins, thereby decreasing their entry into and retention within the subendothelial space of the arterial wall (58).

The role of ApoB-containing lipoproteins in the pathogenesis of cardiovascular disease is illustrated in Figure 1. ApoB-containing lipoproteins are transported across the endothelium primarily via transcytosis, a process tightly regulated by particle size and receptor interactions. Pioneering studies by Simionescu and colleagues demonstrated that particles ≤70 nm in diameter can cross the endothelial barrier and enter the arterial intima, whereas larger particles (>70 nm), such as VLDL and CMs, are effectively excluded because of their size (59, 60). Under atherosclerotic conditions, such as persistent hypercholesterolemia and chronic inflammation, endothelial barrier dysfunction markedly increases vascular permeability and enhances the transcytosis of ApoB-containing lipoproteins (57). After retention within the arterial wall, lipoproteins undergo a series of modifications, including oxidative and enzymatic modifications, as well as aggregation (61). Oxidatively modified lipoproteins can release bioactive molecules, including oxidized phospholipids, which directly activate vascular endothelial cells and trigger pro-inflammatory signaling pathways (62, 63). During the progression of atherosclerotic lesions, activated endothelial cells promote the recruitment and migration of circulating monocytes and lymphocytes into the arterial intima by upregulating key molecules such as vascular cell adhesion molecule-1 (VCAM-1) and monocyte chemoattractant protein-1 (MCP-1). Within the local microenvironment, infiltrating monocytes are further regulated by growth factors secreted by endothelial cells and tissue-resident macrophages and ultimately differentiate into distinct macrophage subsets (64). Macrophages internalize modified lipoproteins through scavenger receptors, such as CD36 and scavenger receptor class A (SR-A), leading to intracellular cholesterol accumulation and foam cell formation (65). Macrophages promote the migration of vascular smooth muscle cells (VSMCs) into the intima and their differentiation by secreting various growth factors and cytokines, particularly platelet-derived growth factor (PDGF). During this process, VSMCs undergo phenotypic switching toward a synthetic phenotype, characterized by increased production of extracellular matrix components, including collagen and elastic fibers, ultimately leading to excessive fibrous tissue deposition (66). Activated VSMCs proliferate and undergo phenotypic switching, leading to increased synthesis of extracellular matrix components and the formation of a fibrous cap over the plaque (67). VSMCs enhance the retention of ApoB-containing lipoproteins by increasing the sulfation and size of glycosaminoglycan (GAG) chains in arterial proteoglycans, thereby accelerating plaque progression (68). During atherosclerosis, degradative enzymes are produced that disrupt the ECM, rendering the fibrous cap susceptible to rupture and potentially triggering thrombosis (69).

**Figure 1:**
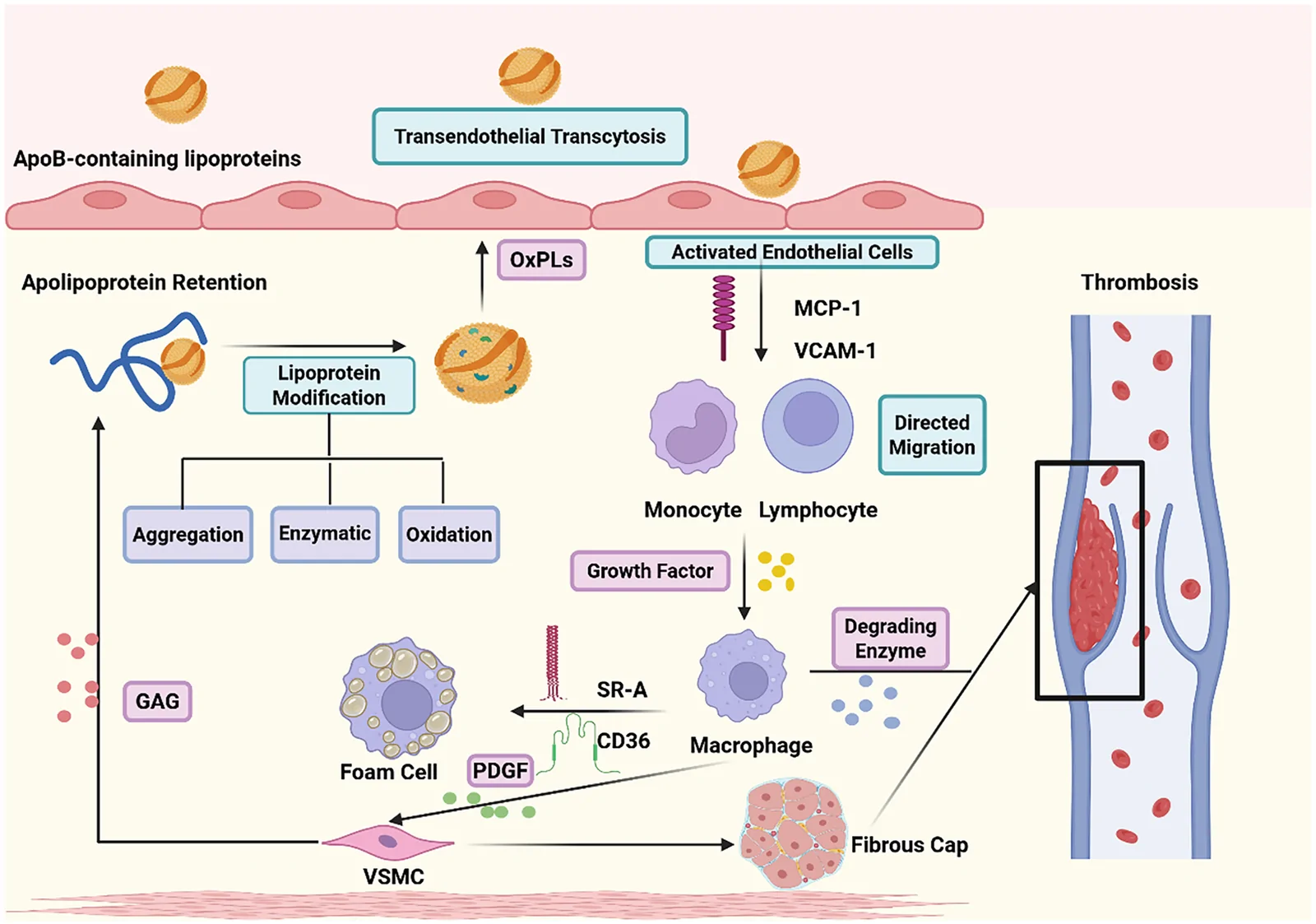
The pathogenesis of cardiovascular diseases caused by apoB (figure created with biorender). VSMC, Vascular smooth muscle cells; OxPLs, oxidized phospholipids; MCP-1, monocyte chemotactic protein-1; VCAM-1, vascular cell adhesion molecule-1; SR-A, scavenger receptor-A; CD36, cluster of differentiation 36; PDGF, particularly platelet-derived growth factor; GAG, glycosaminoglycan.

## 6 Associations of apoB with other clinical conditions

### 6.1 Neurodegenerative diseases

Cardiovascular risk factors are important determinants of Alzheimer's disease (AD) risk. Studies have shown that higher ApoB48 concentrations are associated with a lower risk of AD (70). However, this inverse association should be interpreted with caution. ApoB48 primarily reflects intestinal triglyceride-rich lipoproteins and their remnants and is not equivalent to ApoB, which reflects the overall burden of circulating atherogenic lipoprotein particles. In addition, previous long-term follow-up studies have shown that serum total cholesterol levels may gradually decline for years before clinical diagnosis in individuals who subsequently develop AD, suggesting that metabolic changes during the prodromal phase of neurodegenerative disease may give rise to reverse causation (71). On the other hand, large population-based studies have shown that higher ApoB levels are associated with an increased risk of dementia (72), and Mendelian randomization studies have also suggested that higher ApoB may increase the risk of AD (73).

Therefore, the relationship between ApoB and AD remains unclear. Differences across studies may be related to ApoB isoforms, the timing of measurement, study populations, and potential confounding factors. Gabriel Aumont Rodrigue et al. (74) further explored the potential relationship between ApoB and AD pathology from the perspectives of ApoB expression and lipoprotein metabolism, suggesting that ApoB may be involved in pathological changes across different stages of the disease.

Parkinson's disease (PD) and AD are both common neurodegenerative disorders with complex etiologies that are closely associated with nutritional and metabolic abnormalities. In an observational study, Xu et al. (75) found that higher ApoB levels were associated with a reduced risk of PD. However, the study design does not allow causal inference; therefore, these findings cannot be taken as evidence that ApoB has a protective effect against PD. Longitudinal studies have shown that circulating total cholesterol levels begin to decline during the prodromal phase of PD and fall below expected levels approximately 4 years before clinical diagnosis (76), suggesting that PD-related changes in nutritional status and lipid metabolism may occur before the disease is clinically diagnosed. Accordingly, the observed inverse association between ApoB and PD requires further validation in prospective studies and through genetic causal inference.

### 6.2 Metabolic, microvascular, renal, and neoplastic diseases

Studies have shown that serum ApoB levels and the ApoB/ApoA1 ratio may serve as early negative biomarkers of retinal changes in diabetic macular edema detected by optical coherence tomography (OCT) and optical coherence tomography angiography (OCTA) (77). Dayawa D Agoons et al. (78) analyzed pooled data from 4,888 normoglycemic adults who participated in the National Health and Nutrition Examination Survey (NHANES) from 2007 to 2016. They found that ApoB was not associated with conventional risk factors for diabetes but may be associated with the development of dysglycemia. Rui Shi et al. (79) found that ApoB was independently associated with the presence and severity of diabetic retinopathy (DR), suggesting that strict control of ApoB levels may represent a potential therapeutic strategy for DR. Soie Kwon et al. (80) found that higher ApoB concentrations were associated with an increased risk of end-stage renal disease (ESRD), while the cross-sectional and longitudinal studies by Xu Yu et al. (81) further suggested that serum ApoB showed a particularly strong association with chronic kidney disease (CKD) among multiple lipid measures. In addition, A cohort study found that the ApoB/ApoA-I ratio was an independent prognostic factor for poor outcomes in patients with metastatic colorectal cancer (82). However, most of these studies were observational and primarily demonstrated associations; current evidence is insufficient to establish that lowering ApoB itself improves the incidence or prognosis of these diseases.

### 6.3 Cerebrovascular disease and cognitive impairment

In addition, ApoB levels are associated with the risk of ischemic stroke. Mimi Li et al. (83) demonstrated that a higher ApoB/ApoA-I ratio was associated with intracranial atherosclerotic stenosis (ICAS) in patients with ischemic stroke. Marianne Benn et al. (84) found that homozygosity for the APOB K4154 K variant was associated with a 3- to 5-fold lower risk of ischemic cerebrovascular disease and ischemic stroke. Minwoo Lee et al. (85) found that, during the acute phase of large artery atherosclerotic stroke, the ApoB/ApoA-I ratio independently predicted the development of post-stroke cognitive impairment (PSCI) within 3–6 months after stroke. In addition, a higher ApoB/ApoA-I ratio was specifically associated with impairment in the frontal cognitive domain. Cheng Qian et al. (86) found that the apolipoprotein B/low-density lipoprotein (ApoB/LDL) ratio may serve as a potential clinical indicator for assessing vascular cognitive impairment (VCI). These studies suggest that ApoB-related measures may reflect cerebrovascular atherosclerotic burden and associated neurocognitive outcomes, although their incremental predictive value requires further validation.

### 6.4 Inflammatory diseases

Beyond metabolic and cerebrovascular diseases, ApoB may also be involved in the pathogenesis of inflammatory diseases. Lee Joo Youn et al. (87) found that ApoB enhances inflammatory responses through interactions with enolase-1 (ENO1) expressed on the surface of immune cells, thereby exacerbating rheumatoid arthritis. Overall, the associations between ApoB and a range of diseases beyond CVD suggest that ApoB may be involved in broader metabolic, vascular, and inflammatory processes. However, the strength of evidence varies considerably across disease areas, and most studies have not established causality. Therefore, the pathogenic role of ApoB in ASCVD should not be directly extrapolated to other diseases at present, and its potential value as a biomarker or therapeutic target in non-cardiovascular diseases requires further investigation.

## 7 Clinical value and limitations of apoB

### 7.1 Comparison of apoB with traditional lipid markers

As discussed above, ApoB has clear advantages over conventional lipid measures. Cardiovascular risk is determined primarily by the number of ApoB-containing lipoprotein particles that accumulate within the arterial wall rather than by their cholesterol content. Therefore, the plasma concentration of ApoB-containing particles provides a more direct measure of cardiovascular risk (9, 56). A comparison of ApoB with conventional lipid markers and lipid-based ratios for cardiovascular risk assessment is summarized in Table 2. The 2019 European Society of Cardiology/European Atherosclerosis Society guidelines concluded that ApoB reflects the number of ApoB-containing lipoprotein particles in plasma and is a more accurate marker of the cardiovascular risk associated with these particles. ApoB also reflects the effects of lipid-lowering therapy more accurately than LDL-C or non-HDL-C (33). The AMORIS study suggested that ApoB and ApoA-I may provide additional information for cardiovascular risk assessment, particularly in individuals with dyslipidemia despite normal or low LDL-C concentrations (88). ApoB can be measured using standardized automated assays in modern clinical laboratories. ApoB measurement is more accurate than LDL-C and non-HDL-C, while its turnaround time and cost-effectiveness are comparable to those of routine lipid testing. These assays are readily available in modern clinical chemistry laboratories (89, 90). Using widely available, inexpensive, and standardized methods, ApoB can be measured with greater accuracy, precision, and selectivity than LDL-C and non-HDL-C and should therefore be more widely incorporated into clinical practice (91). In addition, ApoB levels are largely unaffected by fasting status and provide a more accurate assessment than LDL-C in patients with high TG levels or very low LDL-C levels (9, 92, 93).

**Table 2:** Comparison of apoB with conventional lipid markers and lipid-based ratios for cardiovascular risk assessment.

Marker	Biological information	Major limitation	Relative advantages of ApoB	Potential clinical value
ApoB	Atherogenic lipoprotein particle number	Does not indicate cholesterol content per particle	Direct measure of ApoB-containing particle burden	Improved risk stratification, particularly in lipid discordance and metabolic abnormalities
LDL-C	Cholesterol mass within LDL particles	Does not reflect particle number; may be inaccurate with high TG or very low LDL-C	Captures particle number independent of cholesterol content	Identifies residual risk when LDL-C is low but ApoB remains elevated
Non-HDL-C	Cholesterol mass within all non-HDL lipoproteins	Reflects cholesterol mass rather than particle number	More directly quantifies atherogenic particle burden	Useful in hypertriglyceridemia and metabolic syndrome
Total cholesterol	Total plasma cholesterol	Cannot distinguish atherogenic from antiatherogenic lipoproteins	Greater biological specificity for atherogenic particles	More informative for ASCVD risk stratification
ApoB/ApoA-I ratio	Balance between atherogenic and antiatherogenic apolipoproteins	Incremental value over ApoB alone may be limited	ApoB may drive much of the ratio's predictive ability	Useful for comprehensive cardiovascular risk assessment
TC/HDL-C ratio	Relative cholesterol burden	Does not reflect particle number	Direct assessment of atherogenic particle burden	Conventional risk marker with limited mechanistic specificity
LDL-C/HDL-C ratio	Relative LDL-C and HDL-C cholesterol burden	Influenced by HDL-C and does not quantify particles	Better captures atherogenic particle burden	Complementary rather than primary marker

#### 7.1.1 Comparison of apoB and LDL-C

ApoB and LDL-C have distinct physiological implications and predictive value in the assessment of ASCVD risk. LDL-C reflects only the cholesterol content of lipoprotein particles, and its estimation using the Friedewald equation may be inaccurate in patients with hypertriglyceridemia, diabetes, or very low LDL-C levels. Moreover, LDL-C does not reflect differences in lipoprotein particle number or subclasses (94). In contrast, each atherogenic lipoprotein particle contains a single ApoB molecule; therefore, plasma ApoB levels provide a direct and stable measure of the total number of circulating atherogenic particles, independent of variations in their cholesterol content (95). Multiple studies have shown that ApoB outperforms LDL-C in predicting myocardial infarction, CHD, and plaque vulnerability. This advantage is particularly evident in statin-treated patients with residual risk, in whom LDL-C targets have been achieved but the number of atherogenic lipoprotein particles remains elevated; in these patients, ApoB provides more reliable information for risk stratification (92, 96).

#### 7.1.2 Comparison of apoB and non-HDL-C

Non-HDL-C, calculated as total cholesterol minus HDL-C, reflects the total cholesterol content of all atherogenic lipoproteins and correlates well with ApoB levels (97, 98). However, the two measures differ fundamentally in their biological meaning: non-HDL-C remains a measure of cholesterol mass, whereas ApoB directly reflects lipoprotein particle number. Clinical evidence indicates that ApoB provides greater discrimination and more accurate cardiovascular risk prediction than non-HDL-C and can identify high-risk phenotypes characterized by increased numbers of small, dense LDL particles or by an elevated particle burden despite relatively low cholesterol content, which may not be captured by non-HDL-C (99, 100). In women and individuals with hypertriglyceridemia, obesity, or metabolic syndrome, ApoB provides more consistent and robust risk prediction and may overcome the reduced predictive performance of non-HDL-C in these populations (101).

#### 7.1.3 Comparison of apoB and total cholesterol

Total cholesterol, as a conventional lipid measure, reflects only the overall cholesterol concentration in plasma and cannot distinguish atherogenic from protective lipoproteins or capture differences in particle number, size, or subclass composition. ApoB, in contrast, is the core structural protein of atherogenic lipoproteins and directly reflects the total number of atherogenic particles, including VLDL, IDL, LDL, and Lp(a). Accordingly, ApoB has substantially greater pathophysiological relevance and clinical predictive value than total cholesterol (88). Compared with total cholesterol, ApoB may provide an earlier and more accurate indication of atherosclerotic progression. It also offers better risk screening in younger individuals and asymptomatic high-risk populations, helping to overcome the limitations of total cholesterol in cardiovascular risk stratification.

#### 7.1.4 Comparison of the apoB/apoA-I ratio with conventional lipid ratios

The ApoB/ApoA-I ratio integrates the major apolipoproteins of atherogenic and antiatherogenic lipoproteins and therefore reflects the overall balance of lipid metabolism. Its predictive value for CVD is greater than that of conventional lipid ratios, such as total cholesterol/HDL-C and LDL-C/HDL-C (88, 102). The ApoB/ApoA-I ratio has greater sensitivity for predicting premature CHD, acute myocardial infarction, and adverse cardiovascular events, with consistent performance across different ethnic groups, sexes, and age groups. However, after adjustment for traditional risk factors, the independent predictive value of ApoB does not differ significantly from that of the ApoB/ApoA-I ratio. This suggests that ApoB itself is the principal determinant of the risk-predictive ability of this ratio and that measurement of ApoB alone may be sufficient for most clinical risk assessments (103).

### 7.2 Comparison of guidelines

International guidelines increasingly recognize ApoB as a valuable complement or alternative to conventional lipid measures for cardiovascular risk assessment. The 2018 American Heart Association/American College of Cardiology (AHA/ACC) Guideline on the Management of Blood Cholesterol identified ApoB ≥130 mg/dL as a “risk-enhancing factor,” particularly in individuals with TG levels ≥200 mg/dL, to support the initiation or intensification of lipid-lowering therapy (104). The 2019 European Society of Cardiology/European Atherosclerosis Society (ESC/EAS) Guidelines for the Management of Dyslipidaemias identified ApoB as a secondary treatment target, with recommended levels of <65 mg/dL, <80 mg/dL, and <100 mg/dL for patients at very high, high, and moderate cardiovascular risk, respectively. The guidelines also recommend preferential use of ApoB over LDL-C in patients with elevated TG levels (33). The 2021 Canadian Cardiovascular Society guidelines further recommended that, in patients with TG levels >1.5 mmol/L, ApoB or non-HDL-C should be used instead of LDL-C for screening and management (7). More recently, the National Lipid Association (NLA) Expert Clinical Consensus provided more specific guidance on the clinical use of ApoB. The consensus emphasized that ApoB may provide additional information beyond a standard lipid profile, particularly when ApoB and LDL-C are discordant. In such cases, ASCVD risk generally tracks more closely with ApoB or non-HDL-C than with LDL-C. The document also supports the use of ApoB to assist decisions regarding initiation or intensification of lipid-lowering therapy, particularly in patients in whom residual atherogenic lipoprotein burden may not be adequately reflected by LDL-C alone (50). The 2026 ACC/AHA multisociety guideline on the management of dyslipidemia continues to recommend LDL-C and non-HDL-C as the primary treatment targets, while ApoB is used mainly for additional risk assessment in selected populations, particularly in patients who may have residual atherogenic lipoprotein particle burden despite lipid-lowering therapy (105). At present, most guidelines continue to use LDL-C as the primary treatment target, whereas ApoB is used mainly as a complementary marker in selected patients. Compared with ApoB, LDL-C has a longer history of clinical use, and its treatment targets have been more extensively validated in clinical trials and guidelines (40). ApoB and LDL-C are highly correlated in most populations, and the additional value of ApoB is most evident when the two measures are discordant. In the Women's Health Study, the correlation coefficient between ApoB and LDL-C was 0.785, and approximately 18.9% of participants showed discordance. Among these individuals, assessment based on LDL-C alone may underestimate or overestimate CHD risk (106). In addition, ApoB measurement requires an additional laboratory test, which may increase costs and resource burden in resource-limited settings. Therefore, a more practical strategy at present may be to use ApoB selectively in patients with elevated TG levels, metabolic abnormalities, or discordance between ApoB and LDL-C, rather than replacing LDL-C entirely.

### 7.3 Lipid-lowering strategies based on apoB

Changes in ApoB are more sensitive than changes in LDL-C or non-HDL-C for predicting reductions in CHD risk with statin therapy (107). This study provides evidence supporting a strategy of prioritizing the monitoring and targeting of ApoB, thereby favoring a shift from conventional cholesterol-based measures toward ApoB-guided management in lipid-lowering therapy. A randomized, placebo-controlled, double-blind, crossover study found that treatment with simvastatin plus ezetimibe effectively reduced plasma triglyceride-rich lipoprotein (TRL) ApoB48 levels, an effect likely mediated by decreased intestinal secretion of TRL ApoB48 (108). André J Tremblay et al. (109) investigated the effects of ezetimibe on the *in vivo* kinetics of ApoB48 and ApoB100. They found that the reduction in plasma LDL-C levels following ezetimibe treatment was associated with an increased fractional catabolic rate (FCR) of ApoB100-containing lipoproteins.

Studies have shown that the PCSK9 inhibitor evolocumab significantly reduced ApoB levels compared with placebo (110). Mipomersen is an antisense oligonucleotide targeting ApoB mRNA that reduces the production of ApoB-containing lipoproteins by inhibiting ApoB100 synthesis. McGowan et al. showed that adding mipomersen to maximally tolerated lipid-lowering therapy significantly reduced levels of ApoB, LDL-C, total cholesterol, non-HDL-C, and lipoprotein(a) (111). However, mipomersen treatment is associated with adverse effects such as injection-site reactions, elevated liver enzyme levels, and hepatic steatosis, which have substantially limited its clinical use, and the drug has been discontinued in some markets (112). In addition to PCSK9 monoclonal antibodies, inclisiran, a small interfering RNA (siRNA) targeting PCSK9 mRNA in hepatocytes, provides sustained inhibition of PCSK9 synthesis. The ORION trials showed that inclisiran significantly reduced not only LDL-C but also ApoB and non-HDL-C levels (113). In addition, the ATP citrate lyase inhibitor bempedoic acid can further reduce LDL-C, non-HDL-C, and ApoB levels when added to maximally tolerated statin therapy (114). In patients with homozygous familial hypercholesterolemia (HoFH), the microsomal triglyceride transfer protein (MTP) inhibitor lomitapide significantly reduces LDL-C and ApoB levels by inhibiting the assembly and secretion of ApoB-containing lipoproteins (115). In recent years, angiopoietin-like protein 3 (ANGPTL3) has emerged as a novel target for lipid-lowering therapy. Evinacumab, a monoclonal antibody targeting ANGPTL3, reduces atherogenic lipoproteins through a pathway that is relatively independent of the LDL receptor and has shown substantial lipid-lowering effects in patients with HoFH (116). In addition, zodasiran, an RNA interference therapy targeting ANGPTL3, has been shown in phase II clinical studies to reduce ApoB, non-HDL-C, and other atherogenic lipoproteins and remains under clinical development (117). These novel therapeutic strategies suggest that circulating atherogenic lipoprotein particle burden can be further reduced by inhibiting ApoB synthesis, decreasing the assembly of ApoB-containing lipoproteins, or promoting their metabolic clearance.

### 7.4 Issues and challenges

Although multiple studies have demonstrated that ApoB is superior to LDL-C and non-HDL-C for predicting ASCVD risk, it has not yet been widely adopted in routine clinical practice. One barrier to broader use is the lack of consistent guidance for clinicians on how to interpret ApoB results and incorporate them into clinical practice (93). In addition, LDL-C and ApoB show substantially different distributions across populations, such that ApoB levels can vary widely among individuals with the same LDL-C level. This discordance at both the individual and population levels makes it difficult for guidelines worldwide to establish uniform optimal ApoB targets (93, 118).

Although ApoB measurement is already well standardized, some degree of bias may still exist across different assay platforms and reagents. A comparison of three ApoB assays in the MESA study showed that inter-method differences could affect risk classification in some individuals, indicating that further harmonization across measurement systems is still needed (119). At present, randomized controlled trials using ApoB as the primary treatment target and cardiovascular events as the primary outcome are lacking. Current ApoB treatment targets are therefore based mainly on observational studies and analyses of lipid-lowering trials. In addition, ApoB reference ranges may be influenced by ethnic and regional differences, and the applicability of currently recommended thresholds to non-Western populations requires further support from population-based data. Evidence in specific age groups is also relatively limited. Although ApoB levels in childhood show some degree of tracking over time, current evidence is insufficient to support replacing LDL-C with ApoB for routine pediatric screening (120). In older adults, factors such as age and comorbidities may influence the association between ApoB and cardiovascular risk (121).

## 8 Conclusion

ApoB reflects the circulating burden of atherogenic lipoprotein particles and provides complementary information beyond conventional cholesterol-based measures for cardiovascular risk assessment. Evidence from prospective cohort studies, discordance analyses, genetic studies, and clinical guidelines supports the clinical relevance of ApoB, particularly in individuals with elevated triglycerides, metabolic abnormalities, or discordance between ApoB and LDL-C levels. Compared with LDL-C and non-HDL-C, ApoB may better capture variations in atherogenic particle number and residual cardiovascular risk during lipid-lowering therapy. However, several challenges remain before ApoB can be widely incorporated into routine clinical practice. Current guidelines continue to differ regarding the role of ApoB, and LDL-C remains the primary treatment target in many clinical settings because of its extensive evidence base and established therapeutic framework. In addition, the lack of randomized outcome trials using ApoB as the primary treatment target, differences among assay platforms, limited data in specific populations, and uncertainty regarding optimal thresholds remain important issues. Future studies should further evaluate ApoB-guided treatment strategies through prospective clinical trials, establish population-specific reference ranges and treatment targets, and improve the standardization and accessibility of ApoB measurement. With continued accumulation of evidence, ApoB may serve as an important component of personalized cardiovascular risk assessment and lipid management.

## References

[1] Joseph P Leong D McKee M Anand SS Schwalm JD Teo K. Reducing the global burden of cardiovascular disease, part 1: the epidemiology and risk factors. Circulation Rresearch. (2017) 121(6):677–94. 10.1161/CIRCRESAHA.117.308903 28860318

[2] Go AS Mozaffarian D Roger VL Benjamin EJ Berry JD Borden WB. Heart disease and stroke statistics–2013 update: a report from the American Heart Association. Circulation. (2013) 127(1):e6–e245. 10.1161/CIR.0b013e31828124ad 23239837 PMC5408511

[3] Pirillo A Casula M Olmastroni E Norata GD Catapano AL. Global epidemiology of dyslipidaemias. Nat Rev Cardiol. (2021) 18(10):689–700. 10.1038/s41569-021-00541-4 33833450

[4] May HT Nelson JR Lirette ST Kulkarni KR Anderson JL Griswold ME. The utility of the apolipoprotein A1 remnant ratio in predicting incidence coronary heart disease in a primary prevention cohort: the jackson heart study. Eur J Prev Cardiol. (2016) 23(7):769–76. 10.1177/2047487315612733 26481445 PMC5913735

[5] Chermon D Birk R. Polygenic risk, modifiable lifestyle behaviors, and metabolic factors: associations with HDL-C, triglyceride levels, and cardiovascular risk. Nutrients. (2025) 17(13):2244. 10.3390/nu17132244 40647348 PMC12252312

[6] Virani SS Newby LK Arnold SV Bittner V Brewer LPC Demeter SH. 2023 AHA/ACC/ACCP/ASPC/NLA/PCNA guideline for the management of patients with chronic coronary disease: a report of the American Heart Association/American College of Cardiology joint committee on clinical practice guidelines. Circulation. (2023) 148(9):e9–e119. 10.1161/CIR.0000000000001168 37471501

[7] Pearson GJ Thanassoulis G Anderson TJ Barry AR Couture P Dayan N. 2021 Canadian cardiovascular society guidelines for the management of dyslipidemia for the prevention of cardiovascular disease in adults. Can J Cardiol. (2021) 37(8):1129–50. 10.1016/j.cjca.2021.03.016 33781847

[8] Baigent C Blackwell L Emberson J Holland LE Reith C Bhala N. Efficacy and safety of more intensive lowering of LDL cholesterol: a meta-analysis of data from 170,000 participants in 26 randomised trials. Lancet (London, England). (2010) 376(9753):1670–81. 10.1016/S0140-6736(10)61350-5 21067804 PMC2988224

[9] Sniderman AD Thanassoulis G Glavinovic T Navar AM Pencina M Catapano A. Apolipoprotein B particles and cardiovascular disease: a narrative review. JAMA Cardiology. (2019) 4(12):1287–95. 10.1001/jamacardio.2019.3780 31642874 PMC7369156

[10] Johannesen CDL Mortensen MB Langsted A Nordestgaard BG. Apolipoprotein B and non-HDL cholesterol better reflect residual risk than LDL cholesterol in statin-treated patients. J Am Coll Cardiol. (2021) 77(11):1439–50. 10.1016/j.jacc.2021.01.027 33736827

[11] Johannesen CDL Langsted A Nordestgaard BG Mortensen MB. Excess apolipoprotein B and cardiovascular risk in women and men. J Am Coll Cardiol. (2024) 83(23):2262–73. 10.1016/j.jacc.2024.03.423 38839200

[12] Sniderman AD Couture P Martin SS DeGraaf J Lawler PR Cromwell WC. Hypertriglyceridemia and cardiovascular risk: a cautionary note about metabolic confounding. J Lipid Res. (2018) 59(7):1266–75. 10.1194/jlr.R082271 29769239 PMC6027915

[13] Que X Hung M-Y Yeang C Gonen A Prohaska TA Sun X. Publisher correction: oxidized phospholipids are proinflammatory and proatherogenic in hypercholesterolaemic mice. Nature. (2018) 561(7724):E43. 10.1038/s41586-018-0313-x 30013121

[14] Ketelhuth DFJ Rios FJO Wang Y Liu H Johansson ME Fredrikson GN. Identification of a danger-associated peptide from apolipoprotein B100 (ApoBDS-1) that triggers innate proatherogenic responses. Circulation. (2011) 124(22):2433–43:1–7. 10.1161/CIRCULATIONAHA.111.051599 22064596

[15] Shen H Damcott CM Rampersaud E Pollin TI Horenstein RB McArdle PF. Familial defective apolipoprotein B-100 and increased low-density lipoprotein cholesterol and coronary artery calcification in the old order amish. Arch Intern Med. (2010) 170(20):1850–5. 10.1001/archinternmed.2010.384 21059979 PMC3587042

[16] Burnett JR Hooper AJ. Common and rare gene variants affecting plasma LDL cholesterol. The Clinical Biochemist Reviews. (2008) 29(1):11–26. PMID: 18566665. 18566665 PMC2423314

[17] Reimund M Dearborn AD Graziano G Lei H Ciancone AM Kumar A. Structure of apolipoprotein B100 bound to the low-density lipoprotein receptor. Nature. (2025) 638(8051):829–35. 10.1038/s41586-024-08223-0 39663455

[18] Berndsen ZT Cassidy CK. The structure of apolipoprotein B100 from human low-density lipoprotein. Nature. (2025) 638(8051):836–43. 10.1038/s41586-024-08467-w 39662503 PMC11839476

[19] Feingold KR. Introduction to lipids and lipoproteins. [Updated 2024 Jan 14]. In: Feingold KR Adler RA Ahmed SF Anawalt B Blackman MR Chrousos G, editors. Endotext [Internet]. South Dartmouth, MA: MDText.com, Inc (2000).

[20] Nakajima K Nagamine T Fujita MQ Ai M Tanaka A Schaefer E. Apolipoprotein B-48: a unique marker of chylomicron metabolism. In: Makowski G, editors. Advances in Clinical Chemistry. Vol. 64. Amsterdam: Elsevier (2014). p. 117–77. 10.1016/B978-0-12-800263-6.00003-3 24938018

[21] Fazio S Babaev VR Murray AB Hasty AH Carter KJ Gleaves LA. Increased atherosclerosis in mice reconstituted with apolipoprotein E null macrophages. Proc Natl Acad Sci U S A. (1997) 94(9):4647–52. 10.1073/pnas.94.9.4647 9114045 PMC20778

[22] Masuda D Okada T Sairyou M Takafuji K Ohama T Koseki M. Proteomic analysis of human chylomicron remnants isolated by apolipoprotein B-48 immunoprecipitation. J Atheroscler Thromb. (2025) 32(2):226–38. 10.5551/jat.64920 39085140 PMC11802255

[23] Yang C Gu ZW Yang M Gotto AM Jr. Primary structure of apoB-100. Chem Phys Lipids. (1994) 67–68:99–104. 10.1016/0009-3084(94)90128-7 8187250

[24] Blasiole DA Davis RA Attie AD. The physiological and molecular regulation of lipoprotein assembly and secretion. Mol BioSyst. (2007) 3(9):608–19. 10.1039/B700706J 17700861

[25] Hussain MM Rava P Walsh M Rana M Iqbal J. Multiple functions of microsomal triglyceride transfer protein. Nutr Metab (Lond). (2012) 9:14. 10.1186/1743-7075-9-14 22353470 PMC3337244

[26] Koerner CM Roberts BS Neher SB. Endoplasmic reticulum quality control in lipoprotein metabolism. Mol Cell Endocrinol. (2019) 498:110547. 10.1016/j.mce.2019.110547 31442546 PMC6814580

[27] Dashti M Kulik W Hoek F Veerman EC Peppelenbosch MP Rezaee F. A phospholipidomic analysis of all defined human plasma lipoproteins. Sci Rep. (2011) 1:139. 10.1038/srep00139 22355656 PMC3216620

[28] Innerarity TL Young SG Poksay KS Mahley RW Smith RS Milne RW. Structural relationship of human apolipoprotein B48 to apolipoprotein B100. J Clin Invest. (1987) 80(6):1794–8. 10.1172/JCI113273 3680528 PMC442455

[29] Welty FK Lichtenstein AH Barrett PH Dolnikowski GG Schaefer EJ. Human apolipoprotein (apo) B-48 and ApoB-100 kinetics with stable isotopes. Arterioscler, Thromb, Vasc Biol. (1999) 19(12):2966–74. 10.1161/01.ATV.19.12.2966 10591677

[30] Smith D Watts GF Dane-Stewart C Mamo JC. Post-prandial chylomicron response may be predicted by a single measurement of plasma apolipoprotein B48 in the fasting state. Eur J Clin Investig. (1999) 29(3):204–9. 10.1046/j.1365-2362.1999.00431.x 10202376

[31] Masuda D Sakai N Sugimoto T Kitazume-Taneike R Yamashita T Kawase R. Fasting serum apolipoprotein B-48 can be a marker of postprandial hyperlipidemia. J Atheroscler Thromb. (2011) 18(12):1062–70. 10.5551/jat.10470 21946533

[32] Tian J Chen H Liu P Wang C Chen Y. Fasting apolipoprotein B48 is associated with large artery atherosclerotic stroke: a case-control study. Sci Rep. (2019) 9(1):3729. 10.1038/s41598-019-40385-0 30842592 PMC6403240

[33] Mach F Baigent C Catapano AL Koskinas KC Casula M Badimon L. 2019 ESC/EAS guidelines for the management of dyslipidaemias: lipid modification to reduce cardiovascular risk. Eur Heart J. (2020) 41(1):111–88. 10.1093/eurheartj/ehz455 31504418

[34] Correction to. 2018 AHA/ACC/AACVPR/AAPA/ABC/ACPM/ADA/AGS/APhA/ASPC/NLA/PCNA guideline on the management of blood cholesterol: a report of the American College of Cardiology/American Heart Association task force on clinical practice guidelines. Circulation. (2023) 148(7):e5. 10.1161/CIR.0000000000001172 37579012

[35] Chen Z Strack AM Stefanni AC Chen Y Wu W Pan Y. Validation of human ApoB and ApoAI immunoturbidity assays for non-human primate dyslipidemia and atherosclerosis research. J Cardiovasc Transl Res. (2011) 4(3):373–83. 10.1007/s12265-011-9264-4 21394531

[36] Albers JJ Lodge MS Curtiss LK. Evaluation of a monoclonal antibody-based enzyme-linked immunosorbent assay as a candidate reference method for the measurement of apolipoprotein B-100. J Lipid Res. (1989) 30(9):1445–58. 10.1016/S0022-2275(20)38265-1 2600545

[37] Cole J Zubirán R Wolska A Jialal I Remaley AT. Use of apolipoprotein B in the era of precision medicine: time for a paradigm change? J Clin Med. (2023) 12(17):5753. 10.3390/jcm12175737 37685804 PMC10488498

[38] Cobbaert CM Althaus H Begcevic Brkovic I Ceglarek U Coassin S Delatour V. Towards an SI-traceable reference measurement system for seven serum apolipoproteins using bottom-up quantitative proteomics: conceptual approach enabled by cross-disciplinary/cross-sector collaboration. Clin Chem. (2021) 67(3):478–89. 10.1093/clinchem/hvaa239 33331636

[39] Marcovina SM Albers JJ Kennedy H Mei JV Henderson LO Hannon WH. International federation of clinical chemistry standardization project for measurements of apolipoproteins A-I and B. IV. Comparability of apolipoprotein B values by use of international reference material. Clin Chem. (1994) 40(4):586–92. 10.1093/clinchem/40.4.586 8149615

[40] Langlois MR Chapman MJ Cobbaert C Mora S Remaley AT Ros E. Quantifying atherogenic lipoproteins: current and future challenges in the era of personalized medicine and very low concentrations of LDL cholesterol. A consensus statement from EAS and EFLM. Clin Chem. (2018) 64(7):1006–33. 10.1373/clinchem.2018.287037 29760220

[41] Miller WG Myers GL Sakurabayashi I Bachmann LM Caudill SP Dziekonski A. Seven direct methods for measuring HDL and LDL cholesterol compared with ultracentrifugation reference measurement procedures. Clin Chem. (2010) 56(6):977–86. 10.1373/clinchem.2009.142810 20378768 PMC4687457

[42] van Deventer HE Miller WG Myers GL Sakurabayashi I Bachmann LM Caudill SP. Non-HDL cholesterol shows improved accuracy for cardiovascular risk score classification compared to direct or calculated LDL cholesterol in a dyslipidemic population. Clin Chem. (2011) 57(3):490–501. 10.1373/clinchem.2010.154773 21228254 PMC4684263

[43] Avogaro P Bittolo Bon G Avogaro P Cazzolato G Quinci GB. Are apolipoproteins better discriminators than lipids for atherosclerosis? Lancet (London, England). (1979) 1(8122):901–3. 10.1016/S0140-6736(79)91375-8 86668

[44] Sniderman AD Robinson JG. Apob in clinical care: pro and con. Atherosclerosis. (2019) 282:169–75. 10.1016/j.atherosclerosis.2018.11.001 30463677

[45] Wilkins JT Li RC Sniderman A Chan C Lloyd-Jones DM. Discordance between apolipoprotein B and LDL-cholesterol in young adults predicts coronary artery calcification: the CARDIA study. J Am Coll Cardiol. (2016) 67(2):193–201. 10.1016/j.jacc.2015.10.055 26791067 PMC6613392

[46] Sniderman AD Dufresne L Pencina KM Bilgic S Thanassoulis G Pencina MJ. Discordance among apoB, non-high-density lipoprotein cholesterol, and triglycerides: implications for cardiovascular prevention. Eur Heart J. (2024) 45(27):2410–8. 10.1093/eurheartj/ehae258 38700053 PMC11242442

[47] Hwang YC Park CY Ahn HY Cho NH. Prediction of future development of cardiovascular disease with an equation to estimate apolipoprotein B: a community-based cohort study. Medicine (Baltimore). (2016) 95(24):e3644. 10.1097/MD.0000000000003644 27310947 PMC4998433

[48] Zhang C Ni J Chen Z. Apolipoprotein B displays superior predictive value than other lipids for long-term prognosis in coronary atherosclerosis patients and particular subpopulations: a retrospective study. Clin Ther. (2022) 44(8):1071–92. 10.1016/j.clinthera.2022.06.010 35902283

[49] Su X Wang M Zuo Y Wen J Zhai Q Zhang Y. Apolipoprotein particle and cardiovascular risk prediction (from a prospective cohort study). Am J Cardiol. (2023) 201:34–41. 10.1016/j.amjcard.2023.05.052 37352662

[50] Soffer DE Marston NA Maki KC Jacobson TA Bittner VA Peña JM. Role of apolipoprotein B in the clinical management of cardiovascular risk in adults: an expert clinical consensus from the national lipid association. J Clin Lipidol. (2024) 18(5):e647–e63. 10.1016/j.jacl.2024.08.013 39256087 PMC11734832

[51] Li H Wang B Mai Z Yu S Zhou Z Lu H. Paradoxical association between baseline apolipoprotein B and prognosis in coronary artery disease: a 36,460 Chinese cohort study. Front Cardiovasc Med. (2022) 9:822626. 10.3389/fcvm.2022.822626 35146010 PMC8821163

[52] Benn M Nordestgaard BG Jensen GB Tybjaerg-Hansen A. Improving prediction of ischemic cardiovascular disease in the general population using apolipoprotein B: the Copenhagen city heart study. Arterioscler Thromb Vasc Biol. (2007) 27(3):661–70. 10.1161/01.ATV.0000255580.73689.8e 17170368

[53] Zuber V Gill D Ala-Korpela M Langenberg C Butterworth A Bottolo L. High-throughput multivariable Mendelian randomization analysis prioritizes apolipoprotein B as key lipid risk factor for coronary artery disease. Int J Epidemiol. (2021) 50(3):893–901. 10.1093/ije/dyaa216 33130851 PMC8271202

[54] Fu C Liu D Liu Q Wang X Ma X Pan H. Revisiting an old relationship: the causal associations of the ApoB/ApoA1 ratio with cardiometabolic diseases and relative risk factors-a Mendelian randomization analysis. Cardiovasc Diabetol. (2024) 23(1):51. 10.1186/s12933-024-02140-2 38310324 PMC10838437

[55] Richardson TG Sanderson E Palmer TM Ala-Korpela M Ference BA Davey Smith G. Evaluating the relationship between circulating lipoprotein lipids and apolipoproteins with risk of coronary heart disease: a multivariable Mendelian randomisation analysis. PLoS Med. (2020) 17(3):e1003062. 10.1371/journal.pmed.1003062 32203549 PMC7089422

[56] Borén J Williams KJ. The central role of arterial retention of cholesterol-rich apolipoprotein-B-containing lipoproteins in the pathogenesis of atherosclerosis: a triumph of simplicity. Curr Opin Lipidol. (2016) 27(5):473–83. 10.1097/MOL.0000000000000330 27472409

[57] Borén J Packard CJ Binder CJ. Apolipoprotein B-containing lipoproteins in atherogenesis. Nat Rev Cardiol. (2025) 22(6):399–413. 10.1038/s41569-024-01111-0 39743565

[58] Tabas I Williams KJ Borén J. Subendothelial lipoprotein retention as the initiating process in atherosclerosis: update and therapeutic implications. Circulation. (2007) 116(16):1832–44. 10.1161/CIRCULATIONAHA.106.676890 17938300

[59] Simionescu N Simionescu M. Cellular interactions of lipoproteins with the vascular endothelium: endocytosis and transcytosis. Targeted Diagn Ther. (1991) 5:45–95. 10.1201/9780203748831-2 1797171

[60] Vasile E Simionescu M Simionescu N. Visualization of the binding, endocytosis, and transcytosis of low-density lipoprotein in the arterial endothelium in situ. J Cell Biol. (1983) 96(6):1677–89. 10.1083/jcb.96.6.1677 6853599 PMC2112465

[61] Williams KJ Tabas I. The response-to-retention hypothesis of early atherogenesis. Arterioscler, Thromb, Vasc Biol. (1995) 15(5):551–61. 10.1161/01.ATV.15.5.551 7749869 PMC2924812

[62] Bäck M Yurdagul A Jr Tabas I Öörni K Kovanen PT. Inflammation and its resolution in atherosclerosis: mediators and therapeutic opportunities. Nat Rev Cardiol. (2019) 16(7):389–406. 10.1038/s41569-019-0169-2 PMC6727648 30846875

[63] Bäck M Weber C Lutgens E. Regulation of atherosclerotic plaque inflammation. J Intern Med. (2015) 278(5):462–82. 10.1111/joim.12367 25823439

[64] Medrano-Bosch M Simón-Codina B Jiménez W Edelman ER Melgar-Lesmes P. Monocyte-endothelial cell interactions in vascular and tissue remodeling. Front Immunol. (2023) 14:1196033. 10.3389/fimmu.2023.1196033 37483594 PMC10360188

[65] Theofilis P Oikonomou E Tsioufis K Tousoulis D. The role of macrophages in atherosclerosis: pathophysiologic mechanisms and treatment considerations. Int J Mol Sci. (2023) 24(11):9568. 10.3390/ijms24119568 37298518 PMC10253295

[66] Bennett MR Sinha S Owens GK. Vascular smooth muscle cells in atherosclerosis. Circ Res. (2016) 118(4):692–702. 10.1161/CIRCRESAHA.115.306361 26892967 PMC4762053

[67] Jebari-Benslaiman S Galicia-García U Larrea-Sebal A Olaetxea JR Alloza I Vandenbroeck K. Pathophysiology of atherosclerosis. Int J Mol Sci. (2022) 23(6):3346. 10.3390/ijms23063346 35328769 PMC8954705

[68] Kumarapperuma H Chia ZJ Malapitan SM Wight TN Little PJ Kamato D. Response to retention hypothesis as a source of targets for arterial wall-directed therapies to prevent atherosclerosis: a critical review. Atherosclerosis. (2024) 397:118552. 10.1016/j.atherosclerosis.2024.118552 39180958

[69] Libby P. Molecular and cellular mechanisms of the thrombotic complications of atherosclerosis. J Lipid Res. (2009) 50(Suppl):S352–7. 10.1194/jlr.R800099-JLR200 19096046 PMC2674742

[70] Charisis S Lu S Melgarejo JD Satizabal CL Vasan RS Beiser AS. Association of blood lipoprotein levels with incident Alzheimer disease in community-dwelling individuals: the framingham heart study. Neurology. (2025) 104(12):e213715. 10.1212/WNL.0000000000213715 40446198 PMC12129701

[71] Stewart R White LR Xue QL Launer LJ. Twenty-six-year change in total cholesterol levels and incident dementia: the honolulu-Asia aging study. Arch Neurol. (2007) 64(1):103–7. 10.1001/archneur.64.1.103 17210816

[72] Gong J Harris K Peters SAE Woodward M. Serum lipid traits and the risk of dementia: a cohort study of 254,575 women and 214,891 men in the UK biobank. eClinicalMed. (2022) 54:101695. 10.1016/j.eclinm.2022.101695 PMC9561731 36247924

[73] Martin L Boutwell BB Messerlian C Adams CD. Mendelian randomization reveals apolipoprotein B shortens healthspan and possibly increases risk for Alzheimer’s disease. Commun Biol. (2024) 7(1):230. 10.1038/s42003-024-05887-2 38402277 PMC10894226

[74] Aumont-Rodrigue G Picard C Labonté A Poirier J. Apolipoprotein B gene expression and regulation in relation to Alzheimer’s disease pathophysiology. J Lipid Res. (2024) 65(11):100667. 10.1016/j.jlr.2024.100667 39395793 PMC11602985

[75] Xu D-J Shen Y-L Hu M-M Li L-L Fang Y He J-P. Association analyses of nutritional markers with Parkinson’s disease and Alzheimer’s disease. Heliyon. (2024) 10(21):e40191. 10.1016/j.heliyon.2024.e40191 39583797 PMC11584564

[76] Wang K Luo Z Li C Huang X Shiroma EJ Simonsick EM. Blood cholesterol decreases as Parkinson’s disease develops and progresses. Journal of Parkinson’s Disease. (2021) 11(3):1177–86. 10.3233/JPD-212670 PMC12167613 34024785

[77] Soedarman S Julia M Gondhowiardjo T Kurnia KH Prasetya A Triyoga I. Serum apolipoprotein B and B/A1 ratio as early negative biomarkers for OCT- and OCTA-detected retinal changes in diabetic macular edema. Clinical Ophthalmology (Auckland, NZ). (2025) 19:2165–78. 10.2147/OPTH.S527436 PMC12256051 40655034

[78] Agoons DD Agoons BB. Apolipoprotein B and glycemic indices in normoglycemic adults: analysis of the national health and nutrition examination survey, 2007–2016. Cureus. (2025) 17(6):e85656. 10.7759/cureus.85656 40642688 PMC12240877

[79] Shi R Lu Y Liu D Guo Z. Association of serum apolipoprotein B with retinal neurovascular structural alterations in patients with type 2 diabetes: an optical coherence tomography angiography study. Acta Diabetol. (2021) 58(12):1673–81. 10.1007/s00592-021-01775-x 34292395

[80] Kwon S Kim DK Oh K-H Joo KW Lim CS Kim YS. Apolipoprotein B is a risk factor for end-stage renal disease. Clin Kidney J. (2021) 14(2):617–23. 10.1093/ckj/sfz186 33623687 PMC7886579

[81] Xu Y Liu B Lin L Lei F Sun T Zhang X. The association of apolipoprotein B with chronic kidney disease in the Chinese population. Front Endocrinol (Lausanne). (2023) 14:1083614. 10.3389/fendo.2023.1083614 37124758 PMC10132458

[82] Yang DD Chen ZH Wang DS Yu HE Lu JH Xu RH. Prognostic value of the serum apolipoprotein B to apolipoprotein A-I ratio in metastatic colorectal cancer patients. J Cancer. (2020) 11(5):1063–74. 10.7150/jca.35659 31956353 PMC6959062

[83] Li M Ye L Chen C. Association of ApoB/apoA1 ratio with stenosis of intracranial and extracranial arteries in patients with ischaemic stroke. Sci Rep. (2025) 15(1):13622. 10.1038/s41598-025-97625-9 40253439 PMC12009374

[84] Benn M Nordestgaard BG Jensen JS Tybjaerg-Hansen A. Polymorphisms in apolipoprotein B and risk of ischemic stroke. J Clin Endocrinol Metab. (2007) 92(9):3611–7. 10.1210/jc.2007-0221 17595251

[85] Lee M Lim JS Kim Y Park SH Lee SH Kim C. High ApoB/ApoA-I ratio predicts post-stroke cognitive impairment in acute ischemic stroke patients with large artery atherosclerosis. Nutrients. (2023) 15(21):4670. 10.3390/nu15214670 37960323 PMC10648714

[86] Qian C Tan F. Ratio of apoB/LDL: a potential clinical index for vascular cognitive impairment. BMC Neurol. (2016) 16(1):243. 10.1186/s12883-016-0766-1 27887584 PMC5123286

[87] Lee JY Kang MJ Choi JY Park JS Park JK Lee EY. Apolipoprotein B binds to enolase-1 and aggravates inflammation in rheumatoid arthritis. Ann Rheum Dis. (2018) 77(10):1480–9. 10.1136/annrheumdis-2018-213444 29997113

[88] Walldius G Jungner I Holme I Aastveit AH Kolar W Steiner E. High apolipoprotein B, low apolipoprotein A-I, and improvement in the prediction of fatal myocardial infarction (AMORIS study): a prospective study. Lancet. (2001) 358(9298):2026–33. 10.1016/S0140-6736(01)07098-2 11755609

[89] Langlois MR Nordestgaard BG Langsted A Chapman MJ Aakre KM Baum H. Quantifying atherogenic lipoproteins for lipid-lowering strategies: consensus-based recommendations from EAS and EFLM. Clin Chem Lab Med. (2020) 58(4):496–517. 10.1515/cclm-2019-1253 31855562

[90] Kohli-Lynch CN Thanassoulis G Moran AE Sniderman AD. The clinical utility of apoB versus LDL-C/non-HDL-C. Clin Chim Acta. (2020) 508:103–8. 10.1016/j.cca.2020.05.001 32387091

[91] Contois JH Langlois MR Cobbaert C Sniderman AD. Standardization of apolipoprotein B, LDL-cholesterol, and non-HDL-cholesterol. J Am Heart Assoc. (2023) 12(15):e030405. 10.1161/JAHA.123.030405 37489721 PMC10492988

[92] Contois JH McConnell JP Sethi AA Csako G Devaraj S Hoefner DM. Apolipoprotein B and cardiovascular disease risk: position statement from the AACC lipoproteins and vascular diseases division working group on best practices. Clin Chem. (2009) 55(3):407–19. 10.1373/clinchem.2008.118356 19168552

[93] De Oliveira-Gomes D Joshi PH Peterson ED Rohatgi A Khera A Navar AM. Apolipoprotein B: bridging the gap between evidence and clinical practice. Circulation. (2024) 150(1):62–79. 10.1161/CIRCULATIONAHA.124.068885 38950110 PMC11219008

[94] Behbodikhah J Ahmed S Elyasi A Kasselman LJ De Leon J Glass AD. Apolipoprotein B and cardiovascular disease: biomarker and potential therapeutic target. Metabolites. (2021) 11(10):690. 10.3390/metabo11100690 34677405 PMC8540246

[95] Barter PJ Ballantyne CM Carmena R Cabezas M Chapman M Couture P. Apo B versus cholesterol in estimating cardiovascular risk and in guiding therapy: report of the thirty-person/ten-country panel. J Intern Med. (2006) 259(3):247–58. 10.1111/j.1365-2796.2006.01616.x 16476102

[96] Ohwada T Sakamoto T Kanno Y Yokokawa S Amami K Nakazato K. Apolipoprotein B correlates with intra-plaque necrotic core volume in stable coronary artery disease. PLoS One. (2019) 14(2):e0212539. 10.1371/journal.pone.0212539 30779793 PMC6380558

[97] Grundy SM Stone NJ. Elevated apolipoprotein B as a risk-enhancing factor in 2018 cholesterol guidelines. J Clin Lipidol. (2019) 13(3):356–9. 10.1016/j.jacl.2019.05.009 31229022

[98] Sniderman AD Williams K Contois JH Monroe HM McQueen MJ De Graaf J. A meta-analysis of low-density lipoprotein cholesterol, non-high-density lipoprotein cholesterol, and apolipoprotein B as markers of cardiovascular risk. Circ Cardiovasc Qual Outcomes. (2011) 4(3):337–45. 10.1161/CIRCOUTCOMES.110.959247 21487090

[99] Langlois MR Sniderman AD. Non-HDL cholesterol or apoB: which to prefer as a target for the prevention of atherosclerotic cardiovascular disease? Curr Cardiol Rep. (2020) 22(8):67. 10.1007/s11886-020-01323-z 32562186

[100] Holewijn S Den Heijer M Swinkels DW Stalenhoef AF De Graaf J. Apolipoprotein B, non-HDL cholesterol and LDL cholesterol for identifying individuals at increased cardiovascular risk. J Intern Med. (2010) 268(6):567–77. 10.1111/j.1365-2796.2010.02277.x 21091808

[101] Carr SS Hooper AJ Sullivan DR Burnett JR. Non-HDL-cholesterol and apolipoprotein B compared with LDL-cholesterol in atherosclerotic cardiovascular disease risk assessment. Pathology. (2019) 51(2):148–54. 10.1016/j.pathol.2018.11.006 30595507

[102] Tian M Li R Shan Z Wang DW Jiang J Cui G. Comparison of apolipoprotein B/A1 ratio, framingham risk score and TC/HDL-c for predicting clinical outcomes in patients undergoing percutaneous coronary intervention. Lipids Health Dis. (2019) 18(1):202. 10.1186/s12944-019-1144-y 31744496 PMC6864950

[103] Sierra-Johnson J Fisher RM Romero-Corral A Somers VK Lopez-Jimenez F Ohrvik J. Concentration of apolipoprotein B is comparable with the apolipoprotein B/apolipoprotein A-I ratio and better than routine clinical lipid measurements in predicting coronary heart disease mortality: findings from a multi-ethnic US population. Eur Heart J. (2009) 30(6):710–7. 10.1093/eurheartj/ehn347 18676970 PMC2721712

[104] Grundy SM Stone NJ Bailey AL Beam C Birtcher KK Blumenthal RS. 2018 AHA/ACC/AACVPR/AAPA/ABC/ACPM/ADA/AGS/APhA/ASPC/NLA/PCNA guideline on the management of blood cholesterol: a report of the American College of Cardiology/American Heart Association task force on clinical practice Guidelines. Circulation. (2019) 139(25):e1082–e143. 10.1161/CIR.0000000000000625 30586774 PMC7403606

[105] Blumenthal RS Morris PB Gaudino M Johnson HM Anderson TS Bittner VA. 2026 ACC/AHA/AACVPR/ABC/ACPM/ADA/AGS/APhA/ASPC/NLA/PCNA guideline on the management of dyslipidemia: a report of the American College of Cardiology/American Heart Association joint committee on clinical practice guidelines. J Am Coll Cardiol. (2026) 87(19):2624–757. 10.1016/j.jacc.2025.11.016 41824590

[106] Mora S Buring JE Ridker PM. Discordance of low-density lipoprotein (LDL) cholesterol with alternative LDL-related measures and future coronary events. Circulation. (2014) 129(5):553–61. 10.1161/CIRCULATIONAHA.113.005873 24345402 PMC4501252

[107] Thanassoulis G Williams K Ye K Brook R Couture P Lawler PR. Relations of change in plasma levels of LDL-C, non-HDL-C and apoB with risk reduction from statin therapy: a meta-analysis of randomized trials. J Am Heart Assoc. (2014) 3(2):e000759. 10.1161/JAHA.113.000759 24732920 PMC4187506

[108] Tremblay AJ Lamarche B Hogue JC Couture P. Effects of ezetimibe and simvastatin on apolipoprotein B metabolism in males with mixed hyperlipidemia. J Lipid Res. (2009) 50(7):1463–71. 10.1194/jlr.P800061-JLR200 19305020 PMC2694344

[109] Tremblay AJ Lamarche B Cohn JS Hogue JC Couture P. Effect of ezetimibe on the *in vivo* kinetics of apoB-48 and apoB-100 in men with primary hypercholesterolemia. Arterioscler, Thromb, Vasc Biol. (2006) 26(5):1101–6. 10.1161/01.ATV.0000216750.09611.ec 16528005

[110] Toth PP Jones SR Monsalvo ML Elliott-Davey M López JAG Banach M. Effect of evolocumab on non-high-density lipoprotein cholesterol, apolipoprotein B, and lipoprotein(a): a pooled analysis of phase 2 and phase 3 studies. J Am Heart Assoc. (2020) 9(5):e014129. 10.1161/JAHA.119.014129 32114889 PMC7335559

[111] McGowan MP Tardif J-C Ceska R Burgess LJ Soran H Gouni-Berthold I. Randomized, placebo-controlled trial of mipomersen in patients with severe hypercholesterolemia receiving maximally tolerated lipid-lowering therapy. PLoS One. (2012) 7(11):e49006. 10.1371/journal.pone.0049006 23152839 PMC3496741

[112] Alhamadani F Zhang K Parikh R Wu H Rasmussen TP Bahal R. Adverse drug reactions and toxicity of the food and drug administration-approved antisense oligonucleotide drugs. Drug Metab Dispos. (2022) 50(6):879–87. 10.1124/dmd.121.000418 35221289 PMC11022857

[113] Ray KK Kallend D Leiter LA Raal FJ Koenig W Jaros MJ. Effect of inclisiran on lipids in primary prevention: the ORION-11 trial. Eur Heart J. (2022) 43(48):5047–57. 10.1093/eurheartj/ehac615 36331315 PMC9769955

[114] Goldberg AC Leiter LA Stroes ESG Baum SJ Hanselman JC Bloedon LAT. Effect of bempedoic acid vs placebo added to maximally tolerated statins on low-density lipoprotein cholesterol in patients at high risk for cardiovascular disease: the CLEAR wisdom randomized clinical trial. Jama. (2019) 322(18):1780–8. 10.1001/jama.2019.16585 31714986 PMC6865290

[115] Cuchel M Meagher EA Du Toit Theron H Blom DJ Marais AD Hegele RA. Efficacy and safety of a microsomal triglyceride transfer protein inhibitor in patients with homozygous familial hypercholesterolaemia: a single-arm, open-label, phase 3 study. Lancet (London, England). (2013) 381(9860):40–6. 10.1016/S0140-6736(12)61731-0 23122768 PMC4587657

[116] Raal FJ Rosenson RS Reeskamp LF Hovingh GK Kastelein JJP Rubba P. Evinacumab for homozygous familial hypercholesterolemia. N Engl J Med. (2020) 383(8):711–20. 10.1056/NEJMoa2004215 32813947

[117] Rosenson RS Gaudet D Hegele RA Ballantyne CM Nicholls SJ Lucas KJ. Zodasiran, an RNAi therapeutic targeting ANGPTL3, for mixed hyperlipidemia. N Engl J Med. (2024) 391(10):913–25. 10.1056/NEJMoa2404147 38809174

[118] Sayed A Peterson ED Virani SS Sniderman AD Navar AM. Individual variation in the distribution of apolipoprotein B levels across the spectrum of LDL-C or non-HDL-C levels. JAMA Cardiology. (2024) 9(8):741–7. 10.1001/jamacardio.2024.1310 38865115 PMC11170451

[119] Cao J Steffen BT Guan W Remaley AT McConnell JP Palamalai V. A comparison of three apolipoprotein B methods and their associations with incident coronary heart disease risk over a 12-year follow-up period: the multi-ethnic study of atherosclerosis. J Clin Lipidol. (2018) 12(2):300–4. 10.1016/j.jacl.2017.12.013 29370999 PMC12332851

[120] Stanesby O Zhou Z Fonseca R Kidokoro T Otahal P Fraser BJ. Tracking of apolipoprotein B levels measured in childhood and adolescence: systematic review and meta-analysis. Eur J Pediatr. (2024) 183(2):569–80. 10.1007/s00431-023-05350-0 38051379 PMC10912277

[121] Sniderman AD Islam S McQueen M Pencina M Furberg CD Thanassoulis G. Age and cardiovascular risk attributable to apolipoprotein B, low-density lipoprotein cholesterol or non-high-density lipoprotein cholesterol. J Am Heart Assoc. (2016) 5(10):e003665. 10.1161/JAHA.116.003665 27737874 PMC5121475

